# Comparison of Air Conduction and Bone Conduction Masseter Vestibular Evoked Myogenic Potential Between Neurotypical Young Adults and Individuals With Conductive Hearing Loss

**DOI:** 10.7759/cureus.70267

**Published:** 2024-09-26

**Authors:** Bharath Kumar C, Sudharshan Pushpanathan, Yasser Abdullah, Nitish R Patel, Suresh Thontadarya

**Affiliations:** 1 Department of Hearing Studies, Dr. S. R. Chandrasekhar Institute of Speech and Hearing, Bengaluru, IND; 2 Department of Audiology, Shravana Institute of Speech and Hearing, Bellary, IND

**Keywords:** ac vemp, bc vemp, conductive hearing loss, masseter muscle, mvemp, vestibulomasseteric reflex pathway

## Abstract

Introduction

Masseter vestibular evoked myogenic potential (mVEMP) is an acoustically evoked potential recorded from the masseter muscle. It is a recent tool in the vestibular assessment battery that checks the integrity of the vestibulomasseteric reflex pathway, specifically for saccular function.

Need of the study

There is a small pool of subjects with cervical spondylitis and anomalies in the eye muscle where cervical vestibular evoked myogenic potential (cVEMP) and ocular vestibular evoked myogenic potential (oVEMP) tests cannot be performed. In such conditions, mVEMP is considered an appropriate tool to assess the vestibular function. Bone conduction (BC) mVEMP assesses vestibular function in individuals with conductive hearing loss having a significant air-bone gap (ABG). Hence, it is essential to establish a comparative normal range of values for air conduction (AC) and BC mVEMP.

Aim of the study

The study aimed to evaluate the vestibulomasseteric reflex pathway using AC and BC mVEMP for click and 500 Hz tone burst stimuli in neurotypical young adults and compare it to individuals with conductive hearing loss.

Method

Ten neurotypical (20 ears) young adults and five (10 ears) individuals with conductive hearing loss in the age range of 15-40 years without vestibular complaints were recruited for the study. The AC and BC mVEMP were recorded monaurally using click and 500 Hz tone burst stimuli for all the participants.

Results

For neurotypical young adults, the mean latencies of P1 and N1 for AC and BC mVEMP using click were 13.87±1.86 ms and 19.78±2.14 ms and 13.79±2.06 ms and 21.30±1.48 ms, respectively. The P1 and N1 mean latencies for AC and BC mVEMP using 500 Hz tone burst were 14.90±1.50 ms and 21.03±1.30 ms and 14.18±0.95 ms and 19.75±1.71 ms, respectively. For individuals with conductive hearing loss, the P1 and N1 mean latencies for AC and BC mVEMP using click were 15.37±1.46 ms and 20.65±2.65 ms and 15.41±0.68 ms and 21.07±1.45 ms, respectively. AC mVEMP responses were absent for 500 Hz tone burst stimuli. The P1 and N1 mean latencies for BC mVEMP using 500 Hz tone burst stimuli were 14.96±0.6 ms and 22.72±1.42 ms, respectively.

Conclusion

AC mVEMP was absent for 500 Hz tone burst stimulus but present for click stimulus in individuals with conductive hearing loss as there was a larger ABG at the low frequencies than at the mid-high frequencies. BC mVEMP was present for click and 500 Hz tone burst stimuli in all individuals with conductive hearing loss and latencies of P1 and N1 were similar to results obtained for neurotypical young adults and no significant difference was seen.

## Introduction

Vestibular evoked myogenic potentials (VEMPs) are otolith responses that are recorded from certain muscles in the human body when the utricle/saccule or both are stimulated by an acoustic stimulus [1,2]. The most commonly discussed VEMP recordings are cervical VEMP (cVEMP), which is recorded from the sternocleidomastoid (SCM) muscle, and ocular VEMP (oVEMP) recorded from the inferior oblique muscle. A recent addition to these is the masseter vestibular evoked myogenic potential (mVEMP), an acoustically evoked potential recorded from the masseter muscle [3-5]. It is a recent tool in the vestibular assessment battery that checks the integrity of the vestibulomasseteric reflex pathway specifically for saccular function [1,6]. Like other VEMPs, it can be recorded using click and low-frequency tone bursts of air conduction (AC) and bone conduction (BC) stimuli. The literature review suggests that information on the amplitude and latencies of mVEMP recorded for either AC or BC stimuli in neurotypical adults is sporadic. Applications of this technique are also slowly being explored. While cVEMP and oVEMP are clinically very useful, there are a set of clinical populations that cannot undergo these tests, such as those with cervical spondylitis, who have undergone a surgical procedure in the cervical region, and congenital SCM anomalies. Recording of oVEMP from the inferior oblique muscle is impossible in case of an anomaly in the eye muscle due to its location. With the limitations of cVEMP and oVEMP in such cases, mVEMP can be a potentially reliable alternate test [7]. Based on these perspectives, the study aimed to investigate the clinical utility of mVEMP and to assess the vestibulomasseteric reflex pathway by using AC and BC mVEMP for click and 500 Hz tone burst stimuli in neurotypical young adults. Additionally, the study aimed to compare these results with individuals with conductive hearing loss.

Objectives

The objectives of the study are as follows: (1) to estimate and compare the parameters of AC and BC mVEMP (peak latency, peak-to-peak amplitude, and interaural amplitude asymmetry ratio (IAAR)) in neurotypical young adults, (2) to estimate and compare the parameters of AC and BC mVEMP in individuals with conductive hearing loss, and (3) to compare parameters of AC and BC mVEMP between neurotypical adults and individuals with conductive hearing loss.

## Materials and methods

This was a case-control study conducted at the research lab of Dr. S. R. Chandrasekhar Institute of Speech and Hearing, Bengaluru, India. The study was approved by the Institutional Ethics Committee of Bangalore Speech and Hearing Research Foundation (approval number: Bshrf\RC\IEC\IM\IS\03\2024).

Participants

All the patients with conductive hearing loss at the institute's Outpatient Department of Hearing Studies were approached for participation during the study. Information about the purpose, methods, and possible side effects of the study was written in their native language. Only those who gave consent were recruited for the study. The study was carried out from May 3, 2024, to June 28, 2024.

After a detailed case history, all participants underwent tympanometry and pure tone audiometry as routine audiological evaluation. Using 226 Hz probe tone, tympanometry was done using a calibrated Grason-Stadler Inc. TympStar Pro (ANSI S3.9-1987(R2020)) (Eden Prairie, Minnesota, United States), and pure tone audiometry was carried out using a calibrated Grason-Stadler Inc. AudioStar Pro (ANSI S3.6-2018) (Eden Prairie, Minnesota, United States) in a sound-treated room. Ambient noise in the test room was less than the maximum permissible ambient noise sound pressure levels according to ANSI S3.2 (1999).

The participants were divided into group A and group B based on the following criteria: Group A participants had "A" type tympanogram with acoustic reflexes present at 500 Hz, 1 kHz, 2 kHz, and 4 kHz. Hearing sensitivity was within normal limits (the average thresholds of 500 Hz, 1 kHz, and 2 kHz were ≤15 dBHL) with no otological and vestibular symptoms. Ten participants (20 ears) in the age range of 15-40 years were recruited. Group B participants had "B" type or "As" type tympanogram with absent acoustic reflexes at 500 Hz, 1 kHz, 2 kHz, and 4 kHz, bilateral moderate conductive hearing loss with rising configuration, and no active discharge and any vestibular symptoms. Five participants (10 ears) in the age range of 15-40 years were recruited. Pure tone audiometry results indicate that participants had an air-bone gap (ABG) of more than 40 dB till 1 kHz and a gap of less than 20 dB above 2 kHz. Participants with any cognitive and/or psychiatric impairment and a history of loud noise exposure were excluded from the study.

Recording of mVEMP

Preparation

**Figure 1:**
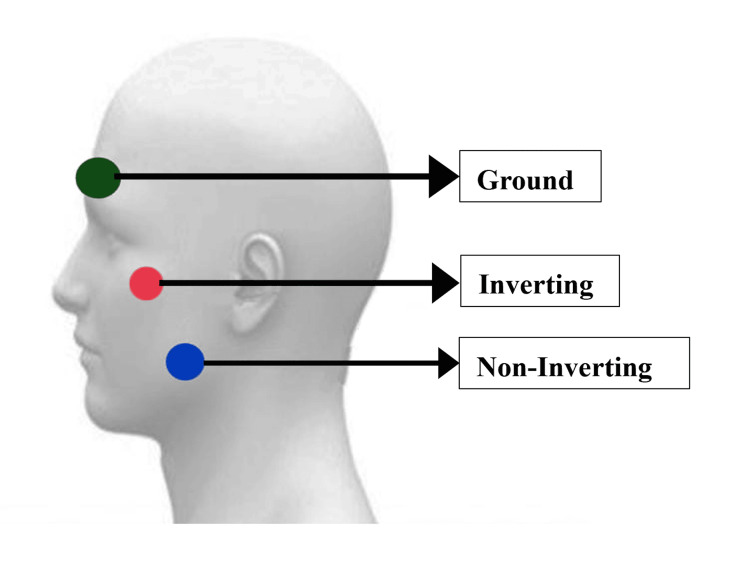
Electrode montage for mVEMP recording mVEMP: masseter vestibular evoked myogenic potential; Ground: lower forehead; Inverting: zygomatic arch; Non-Inverting: lower third of the masseter muscle

The mVEMP was recorded using Intelligent Hearing Systems (IHS) Duet Smart EP (Version 5.54.10) (Miami, Florida, United States) in a sound-treated room. Participants were asked to sit on a chair comfortably with an upright posture. The electrode site (Figure 1) was cleaned using Nuprep brand skin cleansing gel (Weaver and Company, Aurora, Colorado, United States). The electrode was placed using Ten20 conductive gel and surgical tape (Weaver and Company, Aurora, Colorado, United States). During the recording, participants were asked to clench their posterior teeth for the contraction of the masseter muscle and hold it until one recording was completed. A rest period of two minutes was given after each recording to avoid muscle fatigue. The AC mVEMP was recorded using Etymotic Research 3A (ER-3A) insert earphones (Fort Worth, Texas, United States), and the BC mVEMP was recorded using RadioEar B81 bone vibrator (Middelfart, Denmark) placed on the mastoid.

The protocol used in the study for recording mVEMP is given in Table 1 and Table 2.

**Table 1:** Stimulus protocol for AC and BC mVEMP recording AC: air conduction; BC: bone conduction; mVEMP: masseter vestibular evoked myogenic potential; ER-3A: Etymotic Research 3A insert earphones; Hz: hertz

S. no.	Stimulus protocol	AC mVEMP	BC mVEMP
1	Type	Click and 500 Hz tone burst (2-1-2 cycle)	Click and 500 Hz tone burst (2-1-2 cycle)
2	Intensity	95 dBnHL	65 dBnHL
3	Rate	5.1/s	3.1/s
4	Transducers	ER-3A insert earphones	B81 bone vibrator
5	Sweeps	150	150
6	Polarity	Alternation	Alternation
7	Replication	Twice	Twice

**Table 2:** Acquisition protocol for AC and BC mVEMP recording AC: air conduction; BC: bone conduction; mVEMP: masseter vestibular evoked myogenic potential; Hz: hertz; ms: millisecond; kΩ: kiloohms; EMG: electromyography; µV: microvolt

S. no.	Acquisition protocol for AC and BC mVEMP	
1	Filter	High pass: 1.0 Hz; low pass: 1500 Hz
2	Analysis time	75 ms (15 ms pre-stimulus recording)
3	Gain	5000 times
4	Electrode montage	Non-inverting: lower third of masseter muscle; Inverting: zygomatic arch; Ground: lower forehead
5	Electrode impedance	Absolute electrode: <5 kΩ; inter-electrode: <2 kΩ
6	EMG monitoring	50-500 µV
7	Recording	Monaural

Analysis of mVEMP

Two recordings for each stimulus were elicited to check the replicability and reproducibility of the waveform for AC and BC stimuli. Weighted add was taken for the analysis of the responses. The latencies of P1 and N1 were marked and checked by an independent audiologist, and then peak-to-peak amplitude was calculated for each ear. IAAR was calculated for both AC and BC mVEMP responses using the following formula [8]: $IAAR=\left| \frac{\text{Right ear amplitude - Left ear amplitude}}{\text{Right ear amplitude + Left ear amplitude}}\right|\times 100\text{}$.

Statistical analysis

Data were tabulated in an Excel sheet and exported to IBM SPSS Statistics for Windows, Version 20.0 (Released 2011; IBM Corp., Armonk, New York, United States) for statistical analysis. Descriptive statistics were done. The Shapiro-Wilk test was conducted to check whether the data met the normality assumptions. The results indicated that the data satisfied normality conditions. Hence, parametric tests were applied. An independent t-test was performed to assess the significant difference (p<0.05) in the mean scores of each parameter between AC and BC and group A and group B.

## Results

The BC mVEMP responses for click and 500 Hz tone burst and AC mVEMP responses for click were obtained for 100% of the population in the study. However, the AC mVEMP for 500 Hz tone burst was absent in individuals with conductive hearing loss. The mean peak latency, peak-to-peak amplitude, and IAAR were analyzed to interpret the mVEMP responses. 

Objective 1 

Peak latency, peak-to-peak amplitude, and IAAR of AC and BC mVEMP for click and 500 Hz tone burst in neurotypical young adults are shown in Table 3.

**Table 3:** Comparison of mVEMP parameters for neurotypical young adults AC: air conduction; BC: bone conduction; mVEMP: masseter vestibular evoked myogenic potential; SD: standard deviation; P-value: significance level; ms: millisecond; µV: microvolt; IAAR (%): interaural amplitude asymmetry ratio in percentage

Parameters	Click					500 Hz tone burst				
	AC		BC		P-value	AC		BC		P-value
	Mean	SD	Mean	SD		Mean	SD	Mean	SD	
P1 latency (ms)	13.87	1.86	13.79	2.06	0.891	14.9	1.5	14.18	0.95	0.085
N1 latency (ms)	19.78	2.14	21.3	1.48	0.915	21.03	1.3	19.75	1.71	0.269
Amplitude (µV)	91.62	37.17	62.1	21.08	0.160	104.77	41.78	146.32	41.11	0.323
IAAR (%)	23.11	17.71	20.31	13.82	0.725	25.51	11.4	20.36	13.38	0.422

No significant differences were found across the AC and BC recordings for the parameters of mVEMP for click and 500 Hz tone burst stimuli in neurotypical young adults (p>0.05).

**Figure 2:**
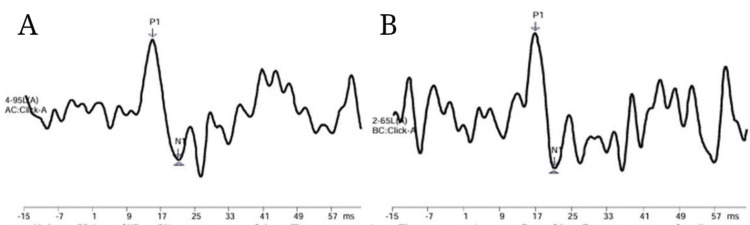
(A) Air conduction mVEMP response using click in neurotypical young adults. (B) Bone conduction mVEMP response using click in neurotypical young adults mVEMP: masseter vestibular evoked myogenic potential

**Figure 3:**
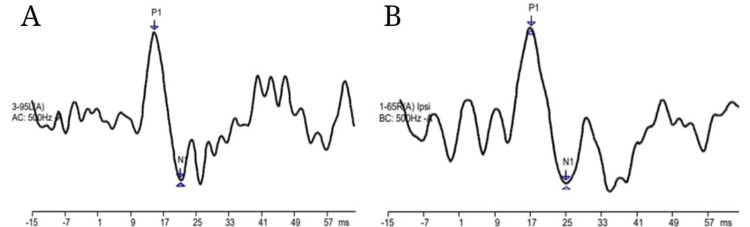
(A) Air conduction mVEMP response using 500 Hz tone burst in neurotypical young adults. (B) Bone conduction mVEMP response using 500 Hz tone burst in neurotypical young adults mVEMP: masseter vestibular evoked myogenic potential

Figure 2 and Figure 3 depict the response waveforms of neurotypical young adults.

Objective 2

Peak latency, peak-to-peak amplitude, and IAAR of AC and BC mVEMP for click and 500 Hz tone burst in individuals with conductive hearing loss are shown in Table 4.

**Table 4:** Comparison of mVEMP parameters for individuals with conductive hearing loss AC: air conduction; BC: bone conduction; mVEMP: masseter vestibular evoked myogenic potential; SD: standard deviation; P-value: significance level; ms: millisecond; µV: microvolt; IAAR (%): interaural amplitude asymmetry ratio in percentage; NA: absent

Parameters	Click					500Hz tone burst			
	AC		BC		P-value	AC		BC	
	Mean	SD	Mean	SD		Mean	SD	Mean	SD
P1 latency (ms)	15.37	1.46	15.41	0.68	0.964	NA	NA	14.96	0.6
N1 latency (ms)	20.65	2.65	21.07	1.45	0.789	NA	NA	22.72	1.42
Amplitude (µV)	64.24	38.36	85.19	27.76	0.437	NA	NA	105.36	31.02
IAAR (%)	32.28	10.4	16.72	12.12	0.303	NA	NA	25.90	8.19

No significant differences were found across the AC and BC recordings for the parameters of mVEMP for click stimuli in individuals with conductive hearing loss (p>0.05). For 500 Hz tone burst stimuli, however, mVEMP responses were absent for AC recordings and present only for BC recordings.

**Figure 4:**
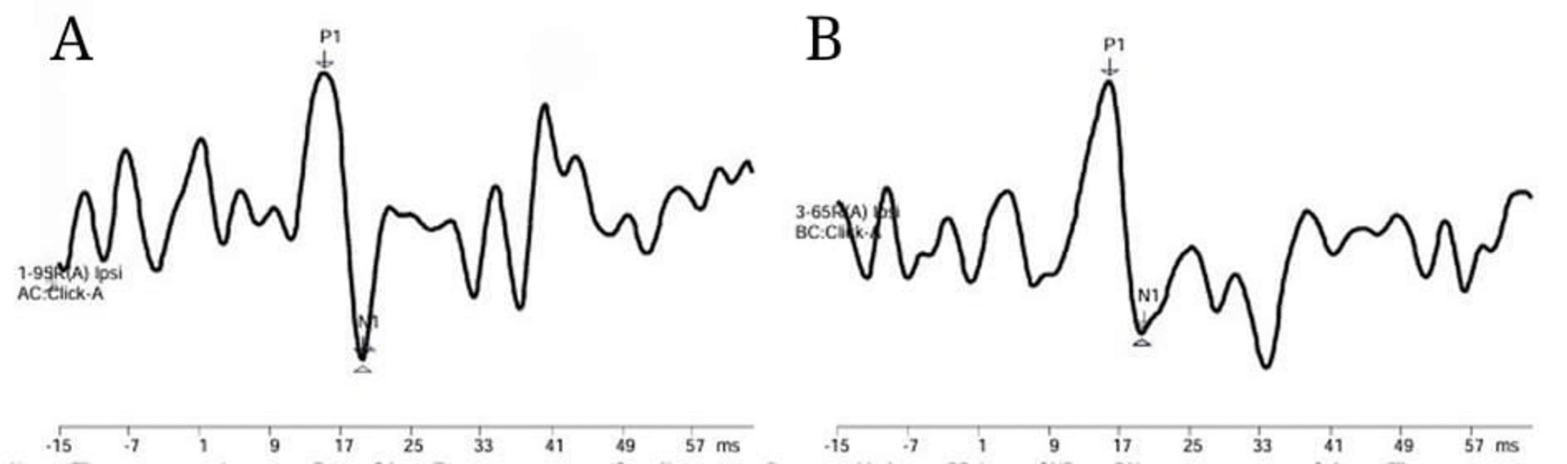
(A) Air conduction mVEMP response using click in individuals with conductive hearing loss. (B) Bone conduction mVEMP response using click in individuals with conductive hearing loss mVEMP: masseter vestibular evoked myogenic potential

**Figure 5:**
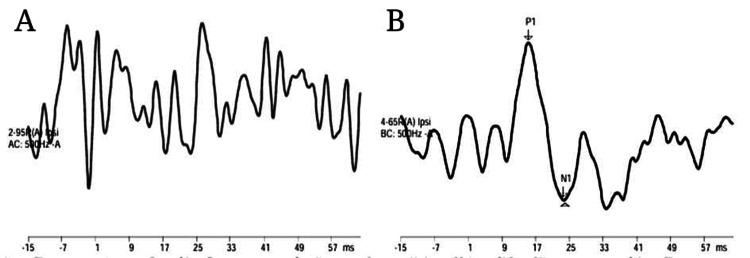
(A) Air conduction mVEMP response using 500 Hz tone burst in individuals with conductive hearing loss. (B) Bone conduction mVEMP response using 500 Hz tone burst in individuals with conductive hearing loss mVEMP: masseter vestibular evoked myogenic potential

Figure 4 and Figure 5 depict the response waveforms of individuals with conductive hearing loss.

Objective 3

No significant differences were found for click and 500 Hz tone burst evoked BC mVEMP parameters and click evoked AC mVEMP across the neurotypical young adults and individuals with conductive hearing loss (p>0.05). There was a significant difference across the parameters of AC mVEMP for 500 Hz tone burst stimuli across the neurotypical young adults and individuals with conductive hearing loss as AC mVEMP was absent for 500 Hz tone burst stimulus (p<0.05).

## Discussion

This study was carried out to explore the clinical utility of BC click and 500 Hz tone burst evoked mVEMP. The study demonstrates that mVEMP can be recorded for BC stimulus in both neurotypical young adults and individuals with conductive hearing loss. The P1 and N1 latencies and peak-to-peak amplitude for click and 500 Hz tone burst evoked BC mVEMP showed responses similar to click and 500 Hz tone burst evoked AC mVEMP in neurotypical young adults. The parameters of the AC mVEMP responses using click and 500 Hz tone burst stimuli between the ears showed no significant differences (p>0.05) in neurotypical young adults, which was similar to the previous studies [1,9-11]. The parameters of BC mVEMP when compared between the ears for click and 500Hz tone burst were similar to the results of the previous studies on AC mVEMP using click and 500 Hz tone burst stimuli in neurotypical young adults [1,9]. The IAAR was less than 40% and showed no significant difference between the click and 500 Hz tone burst evoked AC mVEMP and BC mVEMP (p>0.05) in neurotypical young adults [1,9].

The P1 and N1 latencies and peak-to-peak amplitude for click evoked BC mVEMP showed responses similar to click evoked AC mVEMP in individuals with conductive hearing loss. Individuals with conductive hearing loss showed absent responses for 500 Hz tone burst evoked AC mVEMP but present for click evoked AC mVEMP. This could be possibly explained by the presence of a larger ABG at low-mid frequencies (250 Hz-1 kHz) [12-14]. The presence of click evoked AC mVEMP response can be explained by the frequency response of click stimulus and smaller ABG at mid-high frequencies (2-4 kHz). However, BC mVEMP responses for 500 Hz tone burst stimuli were obtained for individuals with conductive hearing loss as we are bypassing the conductive mechanism. Similar findings were reported in studies conducted on both neurotypical young adults and individuals with conductive hearing loss using BC cVEMP and oVEMP [14-16]. The IAAR was less than 40% and showed no significant difference between click evoked BC mVEMP and AC mVEMP (p>0.05) in individuals with conductive hearing loss, which was similar to the findings of the studies on cVEMP and oVEMP [14-16].

A comparison was made for click evoked AC mVEMP parameters between neurotypical young adults and individuals with conductive hearing loss. No significant differences were observed (p>0.05), which was in agreement with the study's findings on cVEMP [14]. No comparison was made for 500 Hz tone burst evoked AC mVEMP parameters between neurotypical young adults and individuals with conductive hearing loss as 500 Hz tone burst evoked AC mVEMP was absent in individuals with conductive hearing loss. No significant difference was found for click and 500 Hz tone burst evoked BC mVEMP parameters across neurotypical young adults and individuals with conductive hearing loss.

Limitations of the study

However, this study is limited to a smaller number of participants; further studies could be performed with a larger number of individuals for better clinical applicability.

Implications of the study 

The mVEMP is one of the ways to assess the vestibular function in individuals who show contraindications for cVEMP and oVEMP tests, such as those who have cervical spondylitis, who have undergone a surgical procedure in the cervical region, congenital SCM anomalies, and anomalies in the eye muscles. The mVEMP helps in assessing vestibular function in brainstem pathologies like Parkinson's disease, multiple sclerosis, idiopathic random eye movement disorders, and dysarthria. As mVEMP assesses the vestibulomasseteric reflex pathway, it plays a significant role in assessing patients with trigeminal neuralgia. BC mVEMP can be used to evaluate the vestibular function in individuals with conductive hearing loss with a larger ABG.

## Conclusions

The P1 and N1 latencies obtained for AC mVEMP responses using click were clinically within the normal range for both groups. The AC mVEMP responses using click had shorter latencies compared to the 500 Hz tone burst in neurotypical young adults, but this difference was not significant. The AC mVEMP responses were absent for 500 Hz tone burst stimulus but present for click stimulus in individuals with conductive hearing loss as there was a larger ABG at the low frequencies than at the mid-high frequencies. The BC mVEMP normative values for click and 500 Hz tone burst stimuli were established, and no significant difference was seen when compared with AC mVEMP normative values. There was no significant difference in the responses of BC mVEMP in both groups. A variation in peak-to-peak amplitude across the individuals was noticed. The clenching strength of each individual might differ which has resulted in variation in the amplitude.
